# Supplementary material for: University central offices are moving away from doing towards facilitating science communication: A European cross-comparison
Source: PLoS One. 2023 Oct 4;18(10):e0290504. doi: 10.1371/journal.pone.0290504 (PMC10550178; doi:10.1371/journal.pone.0290504)
Supplement: S1 File — (PDF) [file pone.0290504.s008.pdf]

Welcome!

You are invited to participate in this online survey on communication practices of universities.

This study investigates universities' communication with non-specialist audiences – we will refer to this as public communication. Examples of public communication would be maintaining a website/blog/social media for external audiences, organising public events, responding to media enquiries, lobbying decision-makers, and so on. We will ask you questions about the public communication practices of your university, and the resources available for them.

The survey should take no longer than 15-20 minutes to complete. You can interrupt it at any time and return to it later, your answers will be saved. This study is an international collaboration involving the ISCTE - Instituto Universitário de Lisboa (Portugal), the London School of Economics and Political Science (UK), Dusseldorf University (Germany), and Observa - Science in Society (Italy). This research is funded by FCT (Foundation for Science and Technology, Portugal). For more information on the project and team involved, please visit: www.open-pe.com

Your participation in this survey is **voluntary.** You may refuse to take part in the research or exit the survey at any time without penalty. There are no foreseeable risks involved in participating in this study other than those encountered in day-to-day life. All questions are about your communication office practices and resources and not about yourself.

Your responses will remain strictly anonymous. No one will be able to identify you or your answers or your institution, and no one will know whether you or your institution participated in the study.

If you have questions at any time about the study, you may contact the PI of the project, Professor Marta Entradas at marta.entradas@iscte-iul.pt.

We thank you very much for the time you are taking to complete the questionnaire. Your responses are integral to the success of this study.

By clicking on the "Agree" button below you indicate that you voluntarily agree to participate.

☐ Agree

☐ Disagree

Statement of privacy: All responses are strictly confidential.

Block A – Activities and Audiences

We are going to ask you a series of questions about the communications activities your university participates in/organises for external audiences.

[Q1] Could you please let us know whether the following activities are the responsibility of the central communications office where you work, or are the responsibility of other offices within the university, or both.

Within the central communications office	In another office(s) (e.g. research units/departments)	Both within the central comms and others	The university does not do it	I do not know

(1) Writing press releases
(2) Liaising with journalists
(3) Dealing with crisis communications/emergencies
(4) Organising media training for researchers
(5) Managing the university website
(6) Maintaining and monitoring a social media presence
(7) Fundraising
(8) Managing the university brand (logo, brand)
(9) Organising student fairs (welcome, recruitment)
(10) Advertising university events
(11) Cultivating an internal network of contacts at the university
(12) Creating campaigns
(13) Relating to and engaging Alumni
(14) Producing university corporate publications (print and visual)
(15) Feeding research to and facilitating opportunities for academics to engage with policymakers
(16) Networking with groups representing universities
(17) Monitoring education and research policies
(18) Liaising with lobbyists (e.g. consultancies)
(19) Facilitating community engagement in research (e.g. Science shops)
(20) Facilitating university relations with stakeholders (co-creation, knowledge transfer)
(21) Producing research content for the university website
(22) Supporting and encouraging academics to engage with civil society
(23) Organising university public events about research
(24) Assisting academics in grant writing (outreach plans)

[Q2] Based on your previous answers, how efficient do you think this distribution of activities has been at reaching your university's mission and goals?

(1) The university needs more clearly centralised communications
(2) We are doing fine
(3) We need just a bit more coordination
(4) The university needs more clearly decentralised communications
(5) Don't know

[Q3] Distinguishing between communications about education/university activities and communications about research/science activities, how much of the overall public communication do you deal with on a daily basis is about research?

[Slider] [0-100%]

[Q4]. We would like to ask you about your university's preferred means and channels to communicate about research. Roughly, how many times in the past 12 months has the central communications office used the following to disseminate research content to non-specialist audiences?

	Never	Annually (once a year	Quarterly (2-6 times a year)	Monthly (7-20 times a year)	Weekly (> 20 times a year)	Don't know
(1) Public lectures	❍	❍	❍	❍	❍	❍
(2) Public exhibitions	❍	❍	❍	❍	❍	❍
(3) Open Days, guided visits and similar events	❍	❍	❍	❍	❍	❍
(4) Science Festivals/Fairs	❍	❍	❍	❍	❍	❍
(5) National Science Week and similar national events	❍	❍	❍	❍	❍	❍
(6) Science Cafes and similar formats of public discussions events	❍	❍	❍	❍	❍	❍
(7) Citizen science events<br>(8) Policy making events<br>(9) Events with private institutions (industry/business)	❍	❍	❍	❍	❍	❍
(10) Interviews for the media<br>(11) Press conferences	❍	❍	❍	❍	❍	❍
(12) Press releases<br>(13) Articles in magazines/newspapers	❍	❍	❍	❍	❍	❍
(14) Brochures/leaflets/other university publications	❍	❍	❍	❍	❍	❍

[Q5]. Roughly, how many times in the past 12 months has the central communications office used the following new media channels to disseminate research content?

	Never	Quarterly (2-6 times a year)	Monthly (7-20 times a year)	Weekly (> 20 times a year)	Daily	Don't know
(1) Updating the Website	❍	❍	❍	❍	❍	❍
(2) Publishing a new Blog entry	❍	❍	❍	❍	❍	❍
(3) Sharing information on Facebook	❍	❍	❍	❍	❍	❍
(4) Sharing information on Twitter	❍	❍	❍	❍	❍	❍
(5) Publishing a new Podcast/Multimedia video	❍	❍	❍	❍	❍	❍

[Q6]. In the table below you will find a list of non-specialist audiences that you might direct your communications to. How often has the central communications office addressed each of the below audiences in the past 12 months?

	Never	Occasionally	Frequently	Don't know
(1) General public (whoever might be interested)	❍	❍	❍	❍
(2) Prospective students	❍	❍	❍	❍
(3) Members of local municipalities/councils/associations	❍	❍	❍	❍
(4) Delegates from industry	❍	❍	❍	❍
(5) Governments/politicians/policy-makers	❍	❍	❍	❍
(6) Non-governmental organisations (NGOs)	❍	❍	❍	❍
(7) Media and journalists	❍	❍	❍	❍
(8) [Other universities/people like us]	❍	❍	❍	❍

[Q7]. And, have your efforts to reach those audiences increased or decreased over the last 5 years?

	Increased significant ly	Increase d slightly	Stayed the same	Decrease d slightly	Decrease d significant ly	Don't know
(1) General public (whoever might be interested)	❍	❍	❍	❍	❍	❍
(2) Prospective students	❍	❍	❍	❍	❍	❍
(3) Members of local municipalities/councils/associations	❍	❍	❍	❍	❍	❍
(4) Delegates from industry	❍	❍	❍	❍	❍	❍

(5) Governments/politicians/policy-makers	❍	❍	❍	❍	❍	❍
(6) Non-governmental organisations (NGOs)	❍	❍	❍	❍	❍	❍
(7) Media and journalists<br>(8) [Other universities/people like us]	❍	❍	❍	❍	❍	❍

Now we would like to ask you a few questions about the relationships that the central communications office maintain with journalists and the media.

[Q8]. Roughly, how many contacts did the central communications office receive from journalists over the last month?

We received _______ contacts from journalists over the last month

[Q9] Do you maintain a list of journalists and media contacts?

(1) Yes, we have a list/database of journalists
(2) No, we do not have a list, but we have personal contacts
(3) No, we do not have a list/database of journalists

[Q10] When journalists want to contact researchers at your university how do they proceed?

(1) They contact (us) first at the central communications office and we put them in contact with the researchers/departments/schools
(2) Sometimes they contact (us) at the central communications office, other times they contact the researchers/departments/schools directly
(3) They often contact the researchers/departments/schools directly
(4) Don’t know

[Q11] And, how satisfied are you with this management of media relations?

(1) Very satisfied
(2) Satisfied
(3) Neither satisfied nor dissatisfied
(4) Dissatisfied
(5) Very dissatisfied

[Q12]. Over the last 5 years, the number of public communication activities organised/produced by the central communications office has:

(1) Increased significantly
(2) Increased slightly
(3) Stayed the same
(4) Decreased slightly
(5) Decreased significantly
(6) Don't know

Q13. Overall, how successful do you think your communications efforts have been in reaching your university's goals?

(1) Very successful
(2) Successful
(3) Neither successful nor unsuccessful
(4) Unsuccessful
(5) Very unsuccessful
(6) Don't know

Block B – Resources

In the following, we are interested in the resources available at your university for public communications. In particular, how many people are involved in communications tasks and how the activity is resourced.

[Q14] How many people work at the central communications office? This can include people responsible for maintaining the website, social media, organising public events, producing the newsletter, responding to journalists, etc. For simplicity we will refer to them as 'communications staff'. Please count those working ONLY at the central office, and not other communications personnel working at other offices within the university.

There are _____ persons working in the central communications office *(head count)*

[Q15] In the past 5 years, this number has:

(1) Increased significantly
(2) Increased slightly
(3) Stayed the same
(4) Decreased slightly
(5) Decreased significantly
(6) Don't know

[Q16] How many of the 'communications staff' working in the central communications office have research communication tasks in their job description, i.e. dedicate time to tasks related to communicating about research. Please indicate the number.

There are____________ ‘communications staff’ that dedicate time to communicating research content

[Q17] And, How many of the 'communications staff' have a background and/or training in communications? Please count each person **only once** based on their highest degree of specialization.

___________have a post-graduate degree related to communications (Communications/Journalism/Science Communication/Other)
___________have an undergraduate degree related to communications (Communications/Journalism/Science Communication/Other)
___________ have a science communication background (practitioner)
___________ trained on the job

[Q18] How is your university funded? Roughly, what percentage of funds are sourced from recurrent basic funding (e.g. government) and what percentage comes from external sources (e.g. projects/grant funding/industry/business)?

(1) 100% basic funds
(2) 80% basic funds - 20% external funds
(3) 60% basic funds - 40% external funds
(4) 40% basic funds - 60% external funds
(5) 20% basic funds - 80% external funds
(6) 100% external funds
(7) Don't Know

[Q19] Could you please estimate the average **research income** of your university over the last 3 years [(2017+2018+2019) / 3]

________ Millions of euros (€) per year

Q20. Could you please estimate the percentage of the university budget spent on public communications in the last 12 months. This can include actions such as maintenance of the website, printing of brochures, organisation of public events, etc. Please do not consider salaries of the 'communications staff'.

(1) < 1%
(2) 1 - 5%
(3) 6 - 10%
(4) > 10%
(5) Don't Know

Q21. Thinking about the resources devoted to public communications (funding, staff), do you think that your university:

(1) Should devote more resources to public communications
(2) Should devote less resources to public communications
(3) Devotes the right amount of resources to public communications
(4) Don't know

Q22. Please tell us, for each of the following statements about your university's commitment to public communication, whether they are true or false:

	True	False	Don't know
(1) Our university has a public communications strategy			
(2) Our university has a policy encouraging public communication of research			
(3) Our university expects our researchers to be involved in public communications			
(4) Our communication efforts respond to national policies for societal engagement in science			

[Q23]. Roughly, in the last 12 months, what percentage of the academic staff and researchers in your university took part in public communication activities such as public lectures, public debates, media interviews, etc?

(1) < 10%
(2) 10-20%
(3) 20-40%
(4) 40-60%
(5) 60 - 100%
(6) Don´t Know

[Q24] For those academics and researchers who do not usually engage in public communication, what is discouraging them from doing it? Please tell us how much you agree or disagree with the following statements:

	Definitely not true	Unlikely True	Likely true	Very likely true	Don't know
(1) They are not enthusiastic about communicating their work to non-peers					
(2) They do not see public communication as a duty/responsibility					
(3) They do not see benefits for progressing in their careers					
(4) They lack institutional support (e.g. help from staff, training, funding)					
(5) They see public communication as the responsibility of the communications staff					
(6) They do not have time for it					
[7] Our university does not reward academics for their public communication work					

BLOCK C – MESO-CENTRAL RELATIONS

Next, we would like to ask you about the relationships that the central communications office maintains with other offices that have public communication functions. These can be research institutes/departments /schools within your university.

[Q25] The statements below are about the decision-making power about your university's communications strategy. Please choose **the one** that best describes the *modus operandi* at your university:

(1) The central communications office makes the key decisions about the university communication strategy, and research institutes/departments/schools follow
(2) We all work in a common framework but each communications office(s) determines their own communications activities (e.g. follow same guidelines for university image but freely decide on activities)
(3) Research institutes/departments/schools develop their own communication strategy and plan independently from the university framework
(4) All public communications are carried out by the central communications' office

[Q26] How satisfied are you with the distribution of these functions in your university?

(1) Very satisfied
(2) Satisfied
(3) Neither satisfied nor dissatisfied
(4) Dissatisfied
(5) Very dissatisfied

[Q27] Thinking about the relationships between the central communications office and the communications staff working at research institutes/departments/schools, would you agree or disagree that:

(1) Communications offices within the university are duplicating efforts and wasting resources
(2) The university has a good distribution of labour
(3) Communications staff within the university meet regularly to share ideas/best practices

Scale: *(1) Strongly disagree (2) Disagree (3) Neither agree nor disagree (4) Agree (5) Strongly agree*

[Q28] Does the fact that other communications offices in your university deal also with public communication makes your work easier or more difficult in terms of…

(1) Writing a good story becomes…
(2) Sharing interesting research becomes
(3) Sharing what events are going on becomes
(4) Raising funding for communications activities becomes
(5) Harmonizing the institutional message becomes
(6) Standardizing the university's image becomes

Scale: (1) A lot easier (2) Slightly easier (3) Unchanged (4) Slightly more difficult (5) Much more difficult

BLOCK D – RATIONALES/VALUES/PERCEPTIONS

Now we would like to ask you about your university rationales for public communication, perceptions of media coverage and the public, and the expected outcomes of public communication efforts.

[Q29]. How important would you say are the following rationales for your university when deciding about what and with whom to communicate?

(1) To respond to the university's mission
(2) To respond to requirements from funding bodies
(3) To respond to national policies regarding public engagement with science
(4) To raise the university's profile
(5) To attract funding
(6) To give back to taxpayers
(7) To get public support for the university
(8) To disseminate the university's research to the public
(9) To recruit students
(10) To listen and involve the public in the university's activities
(11) To stimulate public debate
(12) To influence policies
(13) To contribute to the legitimacy of science in the public sphere
(14) To further the career of the university staff

Scale: *(1) Very Important • (2) Important • (3) Moderately Important • (4) Slightly Important • (5) Not Important*

Q30. To what extent do you agree or disagree with the following statements about media coverage and dissemination of your research through traditional and new media?

	Strongly disagree	Disagre e	NA nor D	Agr ee	Stron gly agree	DK
(1) Journalists should give more attention to the research conducted at our university						
(2) Journalists have reported badly about our research						
(3) The research done at our university is of little interest to journalists						
[4] News media are the most important channels to disseminate our research						
[5] Social media networks are the most important channels to disseminate our research						

Q31. The following statements express opinions about the public. To what extent do you agree or disagree with each statement?

	Strongly disagree	Disagree	Neither A/D	Agree	Strongly agree	DK
[1] The public is not interested in the research conducted at our university						
[2] The public wants to contribute to science						
[3] We cannot expect a large section of the public to take interest in the research conducted at our university						
[4] We communicate with the public very selectively to avoid trouble						
[5] We would like the public to become more actively involved in decisions about the research conducted at our university						
[6] We would like the public to become more involved in discussing the implications of the research we do, but not necessarily in decisions about our research directions						
[7] The public does not need to be scientifically literate to discuss the implications of our research						

To finalise the questionnaire, we would like to ask you some general questions about your university and yourself. Please note that all information will be kept confidential.

[Q32]. Roughly, how many academic staff work at your university? Please consider all professors and researcher staff; do not count administrative staff.

There are ______academic staff working in this university

[Q33] Roughly, how many students study at your university?

There are _______ students enrolled in this university

[Q34]. Which of the following most closely matches your job title?

(1) Communications staff
(2) Management Staff
(3) Administrative staff
(4) Academic staff
(5) Other (Please specify) ____________________

Q35. How long have you been working in this role?
Selection from a list

Q.00. If you have any comments to the survey, please express them here. (BOX).
